# A Medical Large Language Model-Based System Improves History-Taking Performance Among Medical Students

**DOI:** 10.7759/cureus.114656

**Published:** 2026-08-17

**Authors:** Yuting Huang, Xin Xiao, Xiaoan Sheng, Chao Wang

**Affiliations:** 1 Department of Oncology, Fourth Affiliated Hospital of Anhui Medical University, Hefei, CHN

**Keywords:** artificial intelligence, clinical skills, history-taking, large language model, medical education, medical interview

## Abstract

Introduction: To develop a history-taking training and assessment system based on a medical large language model (MedLLM), evaluate its effect on medical students' history-taking competence, and examine its feasibility and effectiveness as a supplement to conventional interview teaching.

Methods: An intelligent history-taking training and assessment system was built around a domain-fine-tuned Qwen2.5 large language model. Fifty medical students were assigned in equal numbers to an intervention group and a control group (25 each) using a random number table. The intervention group trained with the system, whereas the control group continued to practice through traditional role-play. Outcomes included system acceptance, practice satisfaction, history-taking examination scores, and agreement between manual and automated scoring.

Results: The intervention group rated the system most favorably for feasibility (4.21±0.95) and interest level (3.76±1.14) (P<0.05), and reported significantly greater satisfaction with flexibility (4.06±0.74) and efficiency (4.10±0.60) than the control group (P<0.001). In the manual examination, the intervention group outperformed the control group (84.31±3.92 vs. 80.56±3.41; P<0.05). Overall agreement between automated and manual scoring was good (intraclass correlation coefficient (ICC)=0.76). It was highest for logical organization (ICC=0.87) and clinical reasoning (ICC=0.85) and moderate for communication skills (ICC=0.63) and humanistic care (ICC=0.65).

Conclusion: As a useful complement to traditional teaching, the MedLLM-based history-taking training and assessment system provides a flexible and efficient practice platform that meaningfully strengthens students' history-taking ability. Its automated scoring agrees well with manual assessment, indicating strong potential for clinical skills education.

## Introduction

History-taking is a core clinical competency through which physicians gather patient histories and reach a diagnosis, and its teaching and assessment are central to developing medical students' clinical skills [1]. Effective physician-patient communication not only helps clinicians obtain accurate clinical information but also builds patient trust and strengthens the therapeutic relationship [2]. Yet current medical education still falls short in developing history-taking competence: teaching often emphasizes theory at the expense of practice, leaving students underprepared when they first meet real patients [3]. Traditional history-taking instruction relies largely on one-way lectures, giving students few chances for active participation and hands-on practice [4]. Although several groups [5,6] have reported success with standardized patients (SPs), individual variability and differences in knowledge base limit an SP's ability to reproduce the full range of disease presentations, and the associated cost is high, which constrains widespread adoption [7].

With the rapid progress of artificial intelligence (AI), large language models (LLMs) have shown strong capabilities in language understanding and generation [8]. Applying such models to medical education and to history-taking training in particular may help address current gaps in practical instruction [9]. Compared with conventional approaches, history-taking practice systems built on medical large language models (MedLLMs) offer several advantages [9,10]: they can reproduce a wide range of patient symptoms, provide immediate and objective scoring and feedback, draw on a large repository of disease cases, improve learning efficiency, and reduce teaching costs. We therefore developed an intelligent history-taking training and assessment system based on a MedLLM. The system uses a hierarchical fine-tuning strategy to build a vertical-domain medical LLM and, drawing on a medical case library and SP dialogue corpora, enables the model to convincingly play the role of a patient. Students complete the interview through voice-based interaction with a virtual patient; when the interview ends, the system automatically generates an immediate, objective, and structured evaluation report across multiple dimensions, including history-taking, physician-patient communication, and clinical judgment. Specifically, this study aimed to address three distinct research questions: (1) whether the system is feasible and acceptable to learners; (2) whether exposure to the system as a supplement to traditional instruction is associated with improved history-taking performance compared with conventional practice; and (3) whether the system's automated scoring demonstrates sufficient agreement with expert human scoring to support its use as a formative educational tool. By distinguishing these aspects, we aim to provide a new approach and empirical evidence for AI-enabled medical education and for improving the efficiency and quality of history-taking teaching.

## Materials and methods

System development

The system was developed on the web-based Qwen2.5-72B large model. The medical corpus was compiled from authoritative sources, including standard textbooks, such as Diagnostics and Internal Medicine, and the specialty diagnosis-and-treatment guidelines issued by the National Health Commission. The clinical case library comprised standardized teaching cases from the China Medical Education Case-Sharing Center together with typical specialty cases from the National Clinical Medical Research Center. SP dialogue data were drawn from the MedDialog-CN Chinese medical dialogue dataset and the Hugging Face dataset platform. Using the parameter-efficient LoRA fine-tuning method, we performed domain adaptation of Qwen2.5-72B on the SiliconFlow platform to build a professional-grade vertical medical LLM. During the interview, iFLYTEK SMART-TTS speech-synthesis technology was integrated to support natural dialogue between students and virtual patients: a student's spoken questions were transcribed into text in real time through speech recognition and fed into the system, and the virtual patient's textual replies were converted back into humanlike speech through SMART-TTS. Based on the virtual patient's sex, age, and emotional state, the system automatically matched appropriate timbre and intonation, creating a realistic and fluent conversational experience. The assessment module was built on the Claude-3.5-Sonnet model (Anthropic, San Francisco, CA, USA) and constrained by the history-taking scoring criteria for various diseases used in the standardized residency training examination, together with dedicated prompts; it evaluated interview quality across multiple dimensions, ensuring that results aligned with clinical practice and provided students with objective, reliable feedback. Due to the vast number of disease-specific scenarios (e.g., fever, pneumonia, peptic ulcer), the exact fine-tuning hyperparameters and prompt templates varied dynamically with the assigned disease and are thus too numerous to detail in this manuscript. However, all underlying logic and prompts were strictly anchored to the national standardized residency training grading rubrics.

Interview workflow

**Figure 1:**
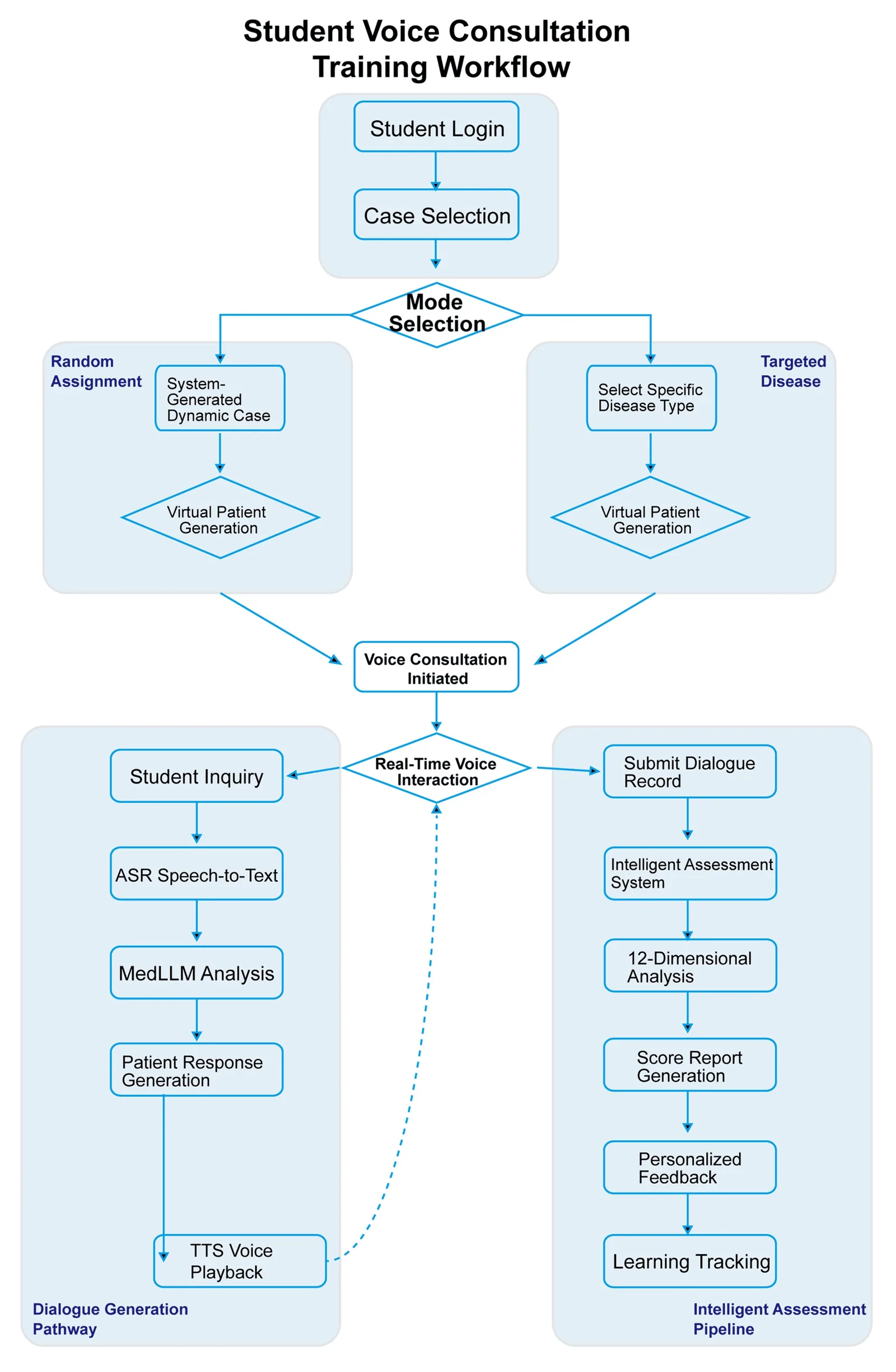
Training workflow of the intelligent history-taking system. ASR: automatic speech recognition; MedLLM: medical large language model; TTS: text-to-speech. The figure was created using Adobe Illustrator 2023 (Adobe Inc., San Jose, CA, USA).

Wearing a headset and microphone, the student logged in and either selected a symptom type or was assigned one at random, after which the system generated a virtual patient and a corresponding clinical scenario. The virtual patient displayed specific disease symptoms, personality traits, and modes of expression, thereby reproducing an authentic consultation setting. The student conducted an interactive interview with the virtual patient by voice, while the system parsed the interview content in real time and generated responses consistent with the case profile. After the interview, the scoring module analyzed the dialogue according to the standardized residency training examination criteria, evaluated the student's performance across dimensions such as completeness, logical organization, and communication skills, and produced a detailed scoring report with suggestions for improvement. All training records were stored automatically, supporting progress tracking and adaptive difficulty adjustment to promote the continued development of history-taking competence. In addition, a novice mode provided real-time cues on possible next lines of inquiry in line with interview standards, and a playback function allowed students to review their own interview dialogues and adjust their learning focus based on the system's data analysis. The detailed workflow is shown in Figure 1.

Study design

This was a prospective, single-center, randomized controlled educational study conducted at the Fourth Affiliated Hospital of Anhui Medical University from September 2024 to June 2025. Fifty medical students undergoing standardized residency training were recruited by convenience sampling and then allocated 1:1 to the intervention or control group (25 each) by simple randomization using a random number table. To ensure allocation concealment, the random sequence generation and participant assignment were executed by an independent research assistant who was not involved in the subsequent teaching or assessment phases.

Study participants

Inclusion criteria: (1) voluntary participation in the study; (2) absence of serious psychological or psychiatric illness; and (3) ability to independently complete the history-taking training and testing.

Exclusion criteria:(1) withdrawal of consent; (2) a diagnosed serious psychological or psychiatric illness; (3) inability to independently complete the training or examination.

Sample size calculation

As this study involved an educational intervention rather than a standard therapeutic clinical trial, the primary outcomes (e.g., manual examination scores) were quantitative continuous data. The sample size was therefore estimated based on a two-sample parallel design. According to our preliminary pilot study, the expected mean difference (δ) in manual scores between the two groups was estimated to be 4 to 5, with a standard deviation (S) ranging from 3 to 4. To ensure a conservative estimate, we adopted the minimum mean difference (δ=4) and the maximum standard deviation (S=4), yielding an effect size (δ/S) of 1. Using the sample size formula for continuous variables from Medical Statistics (4th Edition, edited by Zhenqiu Sun, People's Medical Publishing House, p. 573), with a two-sided significance level (α) of 0.05 and a power (1-β) of 0.90 (β=0.10), the required sample size was calculated to be 22 participants per group. Allowing for a potential attrition or dropout rate of 10% to 20%, the final sample size was expanded to 25 participants per group, for a total of 50 participants.

Confounders

Potential confounders--sex, age, academic performance, duration of residency training, and prior history-taking training experience--were assessed at baseline and compared between groups to confirm comparability.

Teaching and practice arrangements

Both groups received identical theoretical training in history-taking, covering interview techniques and history structure, delivered through lectures and case analysis. During the practice phase, both groups completed one history-taking session per week, one hour each, for eight consecutive weeks. It is crucial to note that while the total practice time was strictly controlled and identical for both groups, the intervention group completed approximately twice the number of clinical cases within that one-hour timeframe. This difference highlights the high efficiency of the AI system, which instantly generated case scenarios, rapidly transcribed dialogues, and provided immediate automated feedback. In contrast, the control group required considerable time for role negotiation, manual scenario setup, and peer-to-peer feedback, resulting in fewer cases completed per hour. All students took a practical examination at the end of the course.

Evaluation indicators

(1) User feedback on the interview system, including usability, rating professionalism, perceived interactivity, feasibility, and interest level; (2) satisfaction with the practice process, including training efficiency, flexibility, physical examination, and scenario simulation; (3) completeness of history content, that is, how thoroughly the chief complaint, present illness, past history, personal history, and family history were elicited; (4) logical organization, that is, the rationality of the questioning sequence and the clarity of reasoning; (5) communication skills, that is, verbal expression, empathy, and effectiveness of guidance; (6) clinical reasoning, that is, targeted questioning, acquisition of key information, and differential-diagnosis ability; (7) humanistic care, that is, communicative attitude, expression of empathy, and psychological support; and (8) overall performance, assessed according to disease-specific scoring criteria.

Assessment methods

(1) A Likert scale was used to survey the intervention group's subjective experience of using the interview system, reflecting their acceptance of it, scored as follows: strongly disagree, 1; disagree, 2; neutral, 3; agree, 4; and strongly agree, 5. (2) Both groups' experience during practice was assessed by a satisfaction survey scored as: very dissatisfied, 1; dissatisfied, 2; neutral, 3; satisfied, 4; and very satisfied, 5. (3) Examination scoring used both automated and manual methods: automated scores were generated by the interview system, and manual scores were assigned by professional examiners according to the scoring criteria, with the total score referenced to disease-specific standards and a maximum of 100 points; in addition, completeness of history content, logical organization, communication skills, clinical reasoning, and humanistic care were each scored out of 10. For manual scoring, the patient in the examination scenario was played by an SP simulating a specific symptom type, and examiners scored both groups after the examination; for automated scoring, the patient was an AI virtual patient, and the system scored both groups on the basis of the contextual content following the interview. During the automated-scoring examination, human examiners simultaneously monitored the dialogue between the student and the virtual patient and again provided a manual score. Manual scoring was conducted by a single, experienced professional examiner for all students to eliminate inter-rater variability. Although the examiner was not blinded to the students' group allocation due to the logistical constraints of the practical examination environment, scoring strictly adhered to the objective structured clinical examination (OSCE) checklists used in the Chinese National Standardized Residency Training program. These rubrics provide highly granular, checklist-based criteria, thereby minimizing subjective bias.

Data collection

(1) User feedback on the interview system was collected from the intervention group; (2) both groups were assessed by human examiners and by the intelligent assessment system, and each student's manual and automated scores were recorded; and (3) during the system-based examination, the system and human examiners scored concurrently, and the human examiners' scores for the students' performance in that examination were also recorded.

Data analysis

(1) The intervention group's acceptance of the interview system was assessed; (2) differences between the intervention and control groups in each practice-phase indicator were compared; (3) between-group differences in human-examiner scores were compared; (4) between-group differences in system scores were compared; and (5) the agreement between human-examiner and system scores was examined.

Statistical analysis

Data were analyzed using SPSS 26.0 (IBM Corp., Armonk, New York, USA) and GraphPad Prism 8. Between-group comparisons of normally distributed continuous variables used the independent samples t-test; comparisons against the neutral value of 3 used the one-sample t test. Categorical data were compared using the χ² test. Effect sizes were reported as Cohen's d for t-tests and φ or Cramér's V for χ² tests. Agreement between scoring methods was assessed with the intraclass correlation coefficient (ICC).

Ethics

The study was approved by the Ethics Committee of the Fourth Affiliated Hospital of Anhui Medical University (Approval No. KYXM-202410-026). All participants provided written informed consent.

## Results

Baseline characteristics

The enrolled residents undergoing standardized training were allocated to the intervention group and the control group (25 each) using a random number table. The intervention group comprised 10 men (40%) and 15 women (60%), with a mean age of 25.16±1.49 years; the control group comprised 12 men (48%) and 13 women (52%), with a mean age of 25.48±1.19 years. The two groups were comparable in sex, age, academic performance, duration of residency training, and prior history-taking training experience, with no statistically significant differences (P>0.05) (Table 1).

**Table 1:** Comparison of baseline characteristics between the two groups.

Characteristic	Intervention (n=25)	Control (n=25)	t/χ²	df	Effect size	P
Sex, n (%)			0.325	1	φ=0.08	0.569
Male	10 (40.0)	12 (48.0)				
Female	15 (60.0)	13 (52.0)				
Age (years)	25.16±1.49	25.48±1.19	-0.837	48	d=-0.24	0.406
Academic performance, n (%)			0.434	2	Cramér's V=0.09	0.805
Average	4 (16.0)	5 (20.0)				
Good	13 (52.0)	14 (56.0)				
Excellent	8 (32.0)	6 (24.0)				
Duration of training (months)	10.44±1.78	10.08±1.47	0.779	48	d=0.22	0.440
Prior interview training, n (%)			0.764	1	φ=0.12	0.382
Yes	17 (68.0)	14 (56.0)				
No	8 (32.0)	11 (44.0)				

User feedback on the interview system

**Figure 2:**
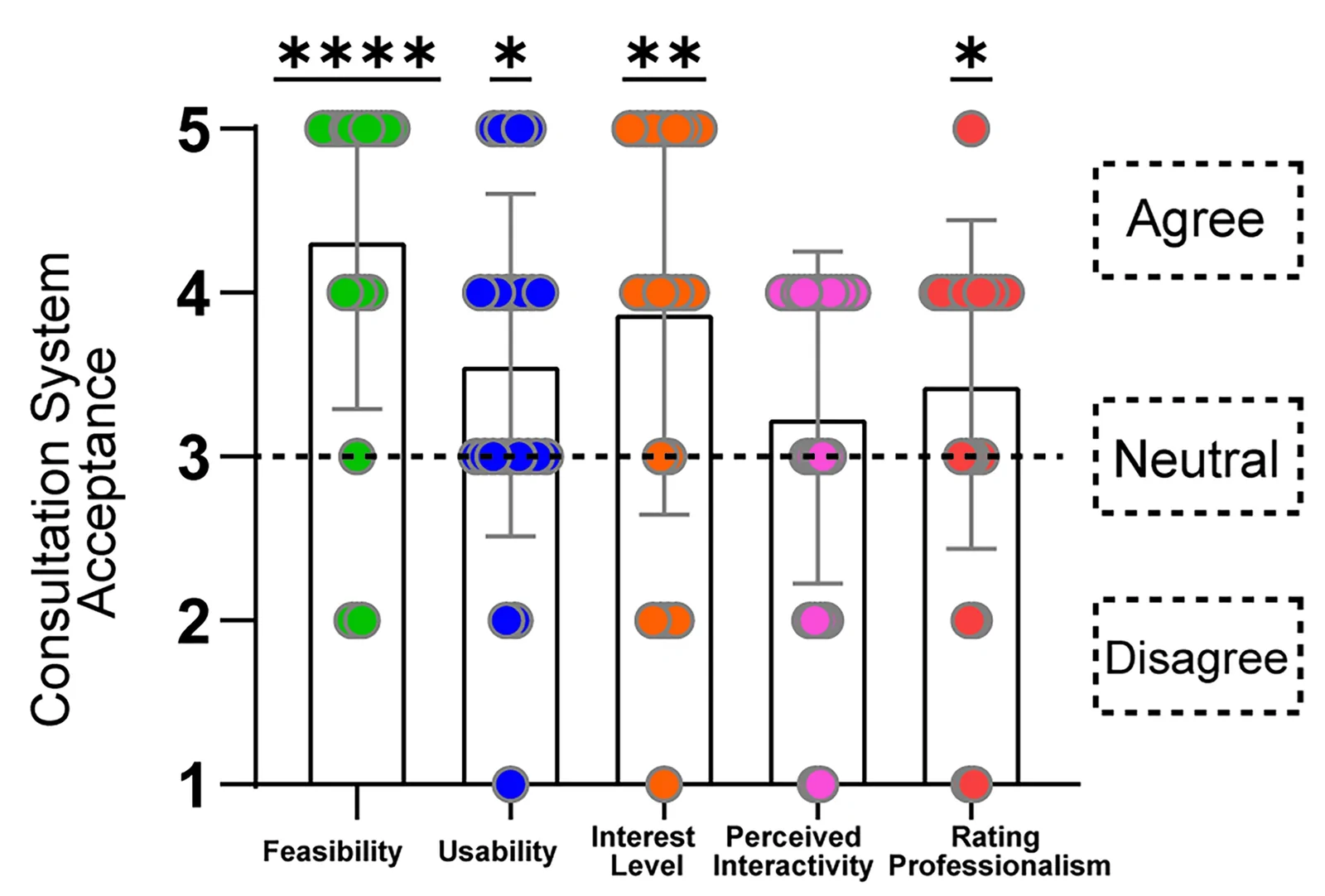
Survey of the intervention group's acceptance of the interview system. Data are expressed as mean ± standard deviation (SD). Statistical analyses were performed using a one-sample t-test comparing the mean scores to a neutral value of 3 (n=25, degrees of freedom (df)=24). The detailed statistical measurements are as follows: feasibility (t=6.370, Cohen's d=1.27, P<0.0001), interest level (t=3.363, Cohen's d=0.67, P=0.003), usability (t=2.682, Cohen's d=0.54, P=0.013), rating professionalism (t=2.478, Cohen's d=0.49, P=0.021), and perceived interactivity (t=1.566, Cohen's d=0.31, P=0.130). *P<0.05, **P<0.01, ****P<0.0001. Graphs were generated using GraphPad Prism 8 (GraphPad Software, San Diego, CA, USA).

A total of 25 questionnaires were distributed to students in the intervention group to assess their acceptance of the interview system, with a 100% valid response rate. Feasibility and interest level received the highest scores (4.21±0.95 and 3.76±1.14, respectively), followed by usability (3.56±1.04) and rating professionalism (3.52±1.06). Compared against the neutral value of 3 using a one-sample t-test (df=24), all these scores differed significantly (all P<0.05). The Perceived Interactivity score [11] was comparatively low (3.34±1.10) and did not differ significantly from the neutral score (P=0.13). The detailed results, including effect sizes (Cohen's d), are shown in Figure 2.

Practice feedback

**Figure 3:**
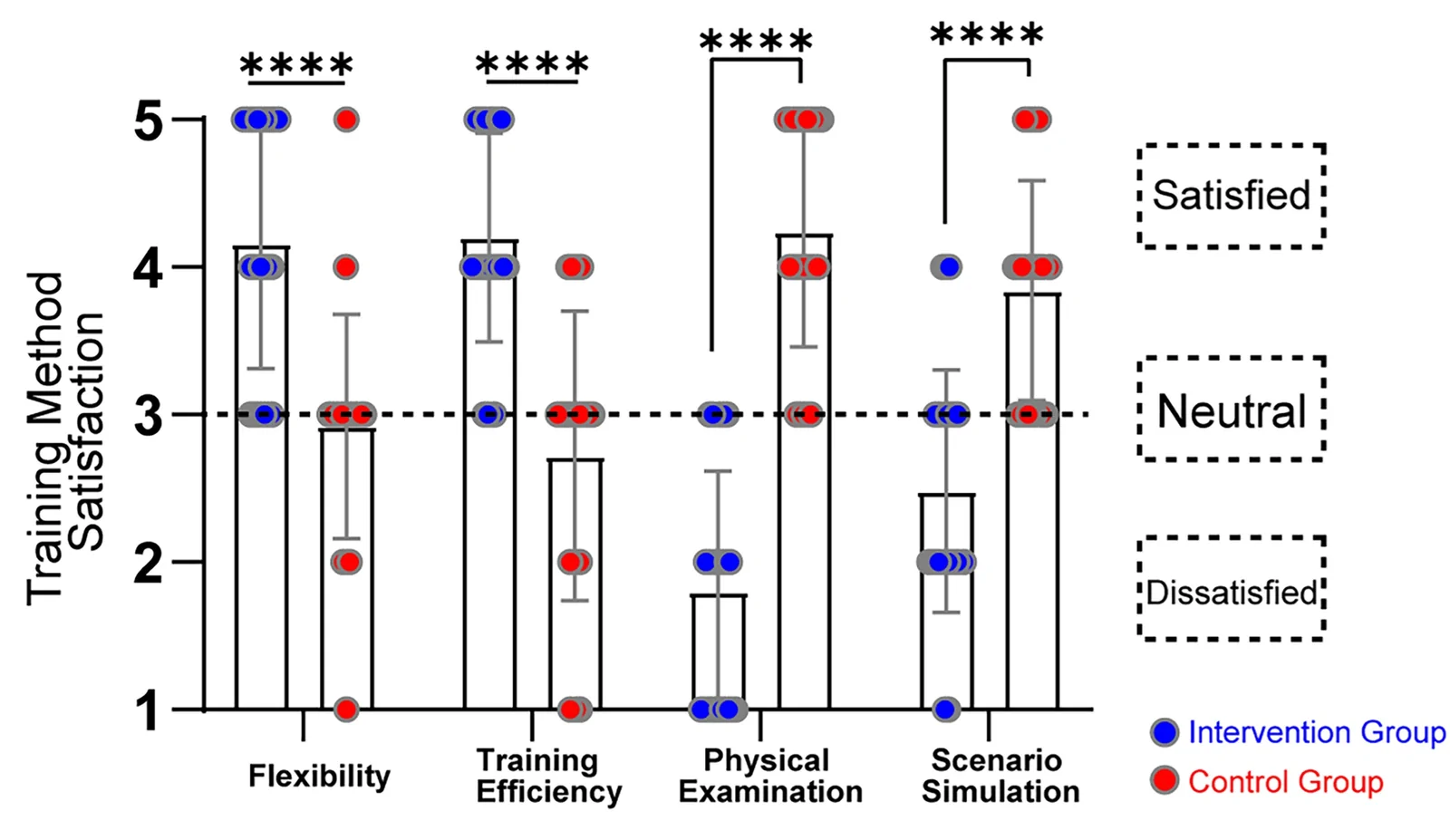
Survey of the two groups' satisfaction with their respective practice modes. Data are expressed as mean ± standard deviation (SD). Statistical comparisons between the intervention group (n=25) and the control group (n=25) were performed using an independent samples t-test (degrees of freedom (df)=48). The detailed statistical measurements are as follows: flexibility (t=4.630, Cohen's d=1.31, P<0.0001), training efficiency (t=5.683, Cohen's d=1.60, P<0.0001), physical examination (t=−10.351, Cohen's d=−2.93, P<0.0001), and scenario simulation (t=−5.981, Cohen's d=−1.70, P<0.0001). ****P<0.0001. Graphs were generated using GraphPad Prism 8 (GraphPad Software, San Diego, CA, USA).

A survey regarding satisfaction with their respective practice modes was distributed to both groups. Based on independent samples t-tests (df=48), the intervention group was significantly more satisfied than the control group regarding flexibility (4.06±0.74 vs. 3.06±0.79; t=4.630, P<0.0001) and training efficiency (4.10±0.60 vs. 2.80±0.98; t=5.683, P<0.0001). Conversely, the control group reported significantly higher satisfaction than the intervention group in physical examination (4.17±0.72 vs. 1.83±0.87; t=−10.351, P<0.0001) and scenario simulation (3.79±0.67 vs. 2.50±0.84; t=−5.981, P<0.0001). The detailed comparisons and effect sizes are presented in Figure 3.

Comparison of scores

Manual examination scores were higher in the intervention group than in the control group (84.31±3.92 vs. 80.56±7.41), a statistically significant difference (P=0.040). System scores were also higher in the intervention group, but the difference was not significant (P=0.116). The intervention group outperformed the control group in logical organization (P=0.011), whereas no significant between-group differences were found in content completeness, communication skills, or humanistic care (Table 2).

**Table 2:** Comparison of scores between the two groups.

Indicator	Intervention (n=25)	Control (n=25)	t	P
Manual score	84.31±3.92	80.56±3.41	2.107	0.040
System score	84.83±3.44	83.30±3.33	1.601	0.116
Content completeness	8.12±0.63	7.92±0.60	1.195	0.238
Logical organization	8.31±0.89	7.68±0.78	2.633	0.011
Communication skills	7.80±0.62	7.94±0.71	-0.766	0.448
Clinical reasoning	8.24±0.65	8.00±0.67	1.288	0.204
Humanistic care	7.84±1.21	7.95±1.03	-0.353	0.726

Concordance between system and manual scoring

During the system-based examination of the 50 trainees, examiners simultaneously provided a second, manual score. The ICC values between system and manual scoring exceeded 0.75 for the total score and for content completeness, logical organization, and clinical reasoning; for communication skills and humanistic care, the values were lower but still exceeded 0.5 (Table 3).

**Table 3:** Comparison of system and manual scoring in the intelligent history-taking examination. Note: ICC: intraclass correlation coefficient; CI: confidence interval; SD: standard deviation; df: degrees of freedom. Criteria for agreement: ICC <0.50, poor; 0.50-0.75, moderate; 0.75-0.90, good; >0.90, excellent.

Item	ICC	95% CI	Mean difference±SD	F (df1, df2)	P
Total score	0.76	0.61-0.85	0.52±2.54	7.29 (49,49)	<0.01
Content completeness	0.79	0.65-0.87	0.03±0.43	8.17 (49,49)	<0.01
Logical organization	0.87	0.78-0.92	0.08±0.46	14.31 (49,49)	<0.01
Communication skills	0.63	0.39-0.78	0.28±0.62	4.98 (49,49)	<0.01
Clinical reasoning	0.85	0.75-0.91	0.07±0.38	12.32 (49,49)	<0.01
Humanistic care	0.65	0.39-0.80	0.42±0.82	5.63 (49,49)	<0.01

## Discussion

Innovations and improvements

As LLMs have advanced rapidly, a growing body of research has introduced them into the medical interview domain [12,13]. The MedLLM-based system developed in this study, built through multidimensional fine-tuning, integrates learning support and automated scoring, and offers several improvements over prior work. First, unlike systems that call general-purpose LLMs directly, ours adopts a hierarchical fine-tuning strategy combining medical domain knowledge, physician-patient dialogue corpora, and standard answers from national examinations, so the virtual patient behaves more realistically and scoring is more accurate. Second, it serves as both a practice and assessment tool, forming a closed "practice-assessment-feedback" loop; because its scoring criteria derive from the reference answers for the history-taking component of the standardized residency training examination, it aligns well with targeted competency training. Third, it provides actionable suggestions to help students strengthen weaknesses. Fourth, it uses a more humanlike text-to-speech (TTS) technology, making the interview smoother and more lifelike. Together, these innovations give the system clear advantages in supporting autonomous practice.

Analysis of results

The intervention group showed high acceptance, with feasibility (4.21) and Interest Level (3.76) scoring significantly above neutral (P<0.05). Holderried et al. [14] reported that 28 students generated 826 question-answer pairs with a generative pre-trained transformer (GPT)-3.5 virtual patient; 97.9% rated credible-consistent with ours and indicating that MedLLM-based systems are well accepted as a supplement to traditional teaching. The Perceived Interactivity score, however, was low (3.34) and did not differ from neutral (P=0.13), reflecting mainly the mechanical voice of the virtual patient and occasional content drift over multiple rounds of questioning. Wang et al. [15] observed similar issues using ChatGPT as an SP, indicating current AI models still have room for improvement. These issues might be addressed through more realistic TTS, better prompt design, or more advanced LLMs. We chose Qwen2.5 mainly for its open fine-tuning pathway, rich medical knowledge, and low hallucination rate, rather than models such as GPT-4, primarily because latency over domestic networks would compromise the experience. This balance between feasibility and user experience is consistent with Temsah et al. [16] on local deployment of DeepSeek R1 in healthcare.

Recently, Holderried et al. [17] applied GPT-4 to the objective structured clinical examination (OSCE) and found the GPT-trained group scored significantly higher than controls (86.79±5.46 vs. 73.64±4.76; P<0.001). Similarly, our intervention group outperformed controls on manual scores (84.31±3.92 vs. 80.56±3.41; P=0.040), especially in logical organization (8.31±0.89 vs. 7.68±0.78; P=0.011). A plausible explanation is that, given the platform's convenience and rapid automated feedback, the intervention group achieved a significantly higher training efficiency, in keeping with the theory of "deliberate practice" [18]: Although both groups were strictly limited to one hour of practice per week with no additional voluntary practice permitted, the high efficiency of the AI system allowed the intervention group to complete more than twice as many clinical scenarios as the controls within that identical timeframe. This increased practice exposure per hour directly translated into improved skills.

Notably, the intervention group scored slightly lower in communication skills (7.80±0.62 vs. 7.94±0.71; P=0.448) and humanistic care (7.84±1.21 vs. 7.95±1.03; P=0.726). Although not significant, these differences point to the limitations of virtual patients in reproducing authentic physician-patient interaction, consistent with Aster et al. [19], who found that virtual simulation cannot fully replace human interaction at the emotional level. Future systems might incorporate richer emotional-expression modes and visual elements, or adopt a blended design combining virtual training with human interaction.

For practice feedback, the intervention group was more satisfied with Flexibility and efficiency (P<0.001), whereas controls scored higher on Physical Examination and Scenario Simulation (P<0.001). This reflects the two-sided nature of the system: it offers a flexible environment but has limitations in reproducing authentic clinical scenarios, particularly physical examination. This shortfall could be partly mitigated by having the virtual patient describe symptom locations verbally; as technology advances, virtual reality could further enhance interactivity [20].

Human-machine comparison

Concordance analysis for the 50 students showed ICC values above 0.75 for the total score and most dimensions, indicating good agreement. The system showed strong capability even on hard-to-quantify dimensions such as logical organization (ICC=0.87) and clinical reasoning (ICC=0.85), a benefit of dual fine-tuning combining medical knowledge with SP dialogue corpora. For communication skills (ICC=0.63) and humanistic care (ICC=0.65), values fell in the moderate-to-good range, reflecting the persistent limitations of AI in assessing subjective abilities involving interpersonal interaction and emotional understanding.

System scoring tended to be more central than manual scoring, with no significant between-group difference (P=0.116), likely reflecting its conservative strategy and limited ability to discriminate subtle differences. Yet this profile makes it well suited as a formative assessment tool [21], giving students objective, stable feedback. The good concordance supports using the system as a "virtual examiner": compared with manual scoring, it offers uniform standards, freedom from mood-related fluctuation, and round-the-clock availability, easing teachers' workload. Such systems hold broad prospects in medical education, particularly in institutions with limited resources.

Limitations and prospects

This study has several important limitations that must be explicitly acknowledged to ensure a balanced interpretation of the findings. First, this was a single-center study with a small convenience sample of only 50 participants, which inherently limits the external validity and generalizability of the results to broader medical student populations. Second, regarding the methodology, due to the logistical constraints of the practical exam, the single human examiner was not blinded to the students' group assignments. While strict adherence to objective OSCE rubrics minimized subjective bias, it could not be completely eliminated. Third, there is a potential confounding effect of differential training exposure; because the AI system was highly efficient, the intervention group completed roughly twice as many cases as the control group within the same allotted 1-hour timeframe. While this highlights the system's efficiency, this higher practice "dose" may partly explain the observed improvements. Fourth, our human-machine concordance analysis revealed only moderate agreement in communication skills and humanistic care, clearly indicating that current AI-based assessments should not be considered equivalent to expert human assessment in evaluating complex, subjective interpersonal dynamics. Finally, the lack of longer-term follow-up prevents us from drawing firm conclusions regarding the retention of these skills or their successful transfer to real clinical encounters with actual patients. Future multicenter studies with larger sample sizes, double-blinded assessments, longitudinal follow-ups, and blended training models are warranted to further validate the role of generative AI in medical education.

## Conclusions

In summary, this study provides credible preliminary evidence that, as a valuable supplement to traditional history-taking instruction, the MedLLM-based system gives medical students a flexible, efficient practice platform. It demonstrates feasibility and potential educational usefulness by improving certain short-term examination outcomes, particularly in logical organization. The good concordance between system and manual scoring supports its promise as a formative assessment tool; however, it does not yet establish interchangeability with expert human assessment, especially for subjective interpersonal dimensions like communication and humanistic care. As AI technology evolves, such systems are likely to play an increasingly important role in medical education, offering a new approach to cultivating clinical history-taking competence. Nonetheless, future large-scale, multicenter studies with double-blinded assessments, standardized practice exposure, and longitudinal follow-up are required to definitively establish its long-term clinical effectiveness and assessment validity.
